# Being overweight in early adulthood is associated with increased mortality in middle age

**DOI:** 10.1038/srep36046

**Published:** 2016-10-26

**Authors:** David Carslake, Mona Jeffreys, George Davey Smith

**Affiliations:** 1 MRC Integrative Epidemiology Unit at the University of Bristol, Oakfield House, Oakfield Grove, Bristol BS8 2BN, UK; 2 School of Social and Community Medicine, University of Bristol, Oakfield House, Oakfield Grove, Bristol BS8 2BN, UK; 3 School of Social and Community Medicine, University of Bristol, Canynge Hall, 39 Whatley Road, Bristol BS8 2PS, UK

## Abstract

Observational analyses of the association between body mass index (BMI) and all-cause mortality often suggest that overweight is neutral or beneficial, but such analyses are potentially confounded by smoking or by reverse causation. The use of BMI measured in early adulthood offers one means of reducing the latter problem. We used a cohort who were first measured while 16–24 year old students at Glasgow University in 1948–1968 and subsequently re-measured in 2000–2003, offering a rare opportunity to compare BMI measured at different ages as a predictor of mortality. Analysis of the later BMI measurements suggested that overweight was beneficial to survival, while analysis of BMI measured in early adulthood suggested that overweight was harmful and that the optimum BMI lay towards the lower end of the recommended range of 18.5–25 kg m^−2^. We interpret the association with later BMI as being probably distorted by reverse causality, although it remains possible instead that the optimum BMI increases with age. Differences when analyses were restricted to healthy non-smokers also suggested some residual confounding by smoking. These results suggest that analyses of BMI recorded in middle or old age probably over-estimate the optimum BMI for survival and should be treated with caution.

High body mass index (BMI) is associated with increased mortality from many causes, particularly cardiovascular disease and some cancers^1^,^2^. The ongoing rises in average BMI shown by many populations are therefore a major cause of concern for population health. There has recently been considerable debate in the scientific and popular literature about the BMI level which is best for health with some studies^3^,^4^,^5^ dividing BMI into categories and finding mortality to be lower in overweight people (BMI of 25–30 kg m^−2^) than in people within the recommended range of 18.5–25 kg m^−2^. If true, this has important clinical and public health implications. However, observational studies of the association between BMI and mortality are vulnerable to confounding, from two sources in particular. First, smoking is known to lower BMI, and its association with mortality from respiratory disease and many cancers is well-established. Secondly, certain medical conditions may cause subjects to lose or not gain weight with age, as well as predisposing them to mortality. In the absence of rigorous randomised trials, several methods have been used to adjust, at least in part, for this confounding. Smoking behaviour may be reported and included as a covariate, although such adjustment can never allow fully for individual differences in smoking behaviour^6^,^7^,^8^. Genetic or other instrumental variables offer a way around unmeasured confounding, but the estimates they give are often imprecise, and rely on several assumptions^9^. The role of confounding by smoking behaviour may be investigated by breaking total mortality down into specific causes; particularly comparing those causes of death known to be associated with smoking with those not so associated. To reduce confounding by pre-existing disease, the first few years of follow-up following the measurement of BMI are sometimes excluded (e.g. refs 1 and 10 but see ref. 11). Here, we use a variant of this approach by using BMI measured in early adulthood (students at the University of Glasgow between 1948 and 1968). We compare the results with estimates made using a subset of the study cohort resampled in middle age.

## Results

**Figure 1:**
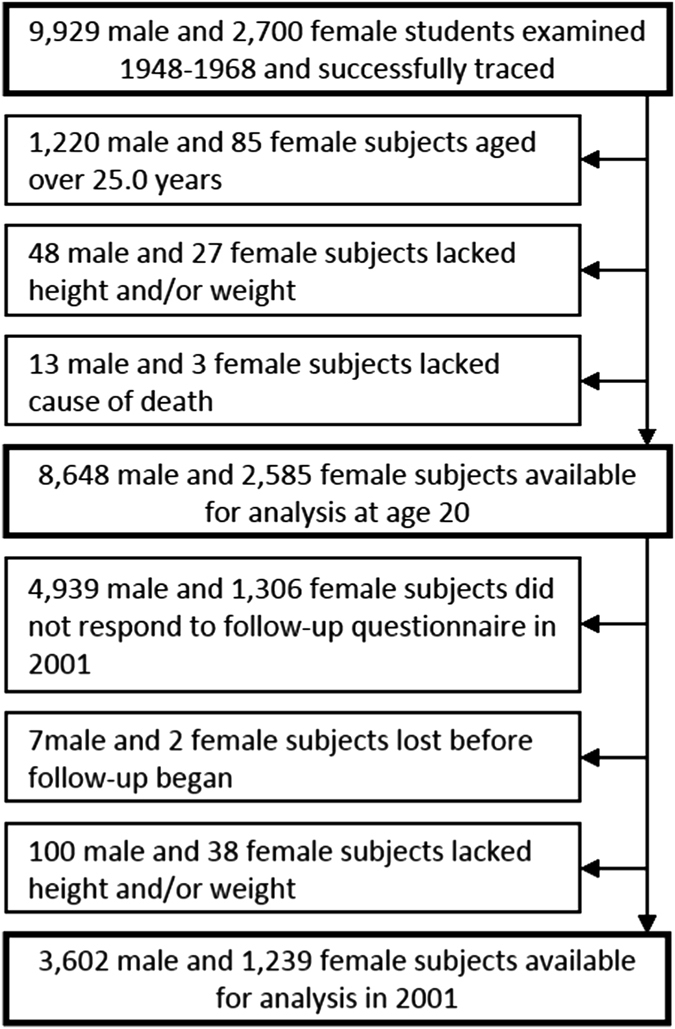
Flow of participants through the study.

9,929 male and 2,700 female students from the Glasgow Alumni Cohort were successfully traced. Exclusions and final sample sizes for each analysis are shown in Fig. 1. Of the 8,648 men and 2,585 women available for the analysis of BMI at age 20, 572 men (6.6%) and 243 women (9.4%) were underweight, 7,547 men (87.3%) and 2,161 women (83.6%) were recommended weight, 497 men (5.7%) and 173 women (6.7%) were overweight and 32 men (0.4%) and 8 women (0.3%) were obese. Baseline characteristics of subjects according to quartiles of BMI are shown in Table 1 (for BMI at age 20) and Supplementary Table S2 (for BMI in 2001). Women were only slightly over-represented in the highest and lowest quartiles of BMI at age 20, but comprised 38% of those in the lowest quartile in 2001, despite being only 26% of the sample at this time. Participants with higher BMI at age 20 tended to be older and shorter and had a lower pulse rate at age 20, came from larger families and were less likely to be the oldest of their siblings. Those in the third quartile of BMI were slightly less likely to smoke, but the difference was small and the linear association could not be distinguished from the null. There was no clear association with paternal SEP. Participants with the highest BMI at age 20 also had high BMI in 2001 (BMI in 2001 increased by 0.56 (95% confidence interval (CI): 0.52, 0.60) kg m^−2^ per kg m^−2^ at age 20). They tended to be younger and were more likely to smoke in 2001. Non-smokers at age 20 with higher BMI were more likely to have taken up (and in some cases, subsequently quit) smoking by 2001, and smokers with high BMI were less likely to have given up.

Participants with the highest and lowest BMI in 2001 came from slightly smaller families on average than those with intermediate BMI and were thus more likely to be the eldest child. Smoking in 2001 was more common among the lowest quartile of BMI. Those with higher BMI in 2001 were more likely to have taken up smoking (if non-smokers at age 20), while those in the lowest quartile of BMI in 2001 were less likely to have given up smoking (if smokers at age 20). High paternal SEP was positively associated with smoking at age 20 (risk ratio 1.15; 95% CI: 1.08, 1.22) and negatively associated with smoking in 2001 (risk ratio 0.91; 95% CI: 0.76, 1.09).

**Figure 2:**
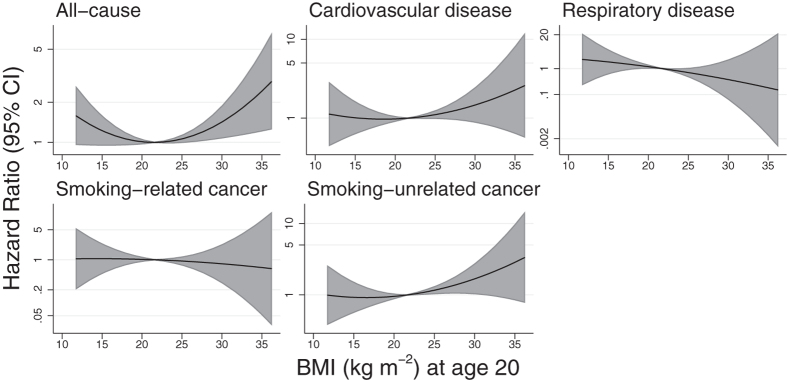
Fitted hazard ratios for all-cause and cause-specific mortality from the quadratic model of BMI at age 20. The midpoint of the recommended BMI range (18.5–25 kg m^−2^) is used as the reference point. Analyses were adjusted for sex, date of birth (cubic splines) and smoking behaviour at age 20.

Analyses conducted separately on men and women gave similar results (Supplementary Tables S4 and S5; P > 0.05 in all heterogeneity tests), although the power was low when women were tested alone. Only the combined analyses will be considered further. Table 2 shows hazard ratios (HR) per 5 kg m^−2^ of BMI at age 20. Participants with a higher BMI in early adulthood were at increased risk of subsequent mortality from cardiovascular disease (HR per 5 kg m^−2^: 1.13; 95% CI: 0.96, 1.32). There was a strong negative association between BMI in early adulthood and mortality from (non-malignant) respiratory diseases (HR per 5 kg m^−2^: 0.61; 95% CI: 0.42, 0.87). High BMI in early adulthood was positively associated with mortality from those cancers not associated with smoking, and weakly negatively associated with smoking-related cancers. The linear association of all-cause mortality with BMI was close to the null, but there was evidence from the quadratic models (Table 2, Fig. 2) of nonlinearity in the association with BMI at age 20, with predicted minimal mortality at 21.5 kg m^−2^ (95% CI: 16.7, 23.7) and increased mortality at higher and lower BMI. There was no evidence for quadratic nonlinearity for any specific cause of death. Results without adjustment for smoking behaviour (Supplementary Table S3) were very similar. There was no substantial evidence for departure from the proportional hazards assumption (P > 0.05 for all causes of death).

Because of the rarity of subjects with extremes of BMI at age 20, the midpoints of all four quartiles fell within the recommended range of BMI. Plots of HR by these quartiles (Supplementary Fig. S1) suggested that these associations were largely linear, including that for all-cause mortality. However, the analyses by the conventional categories of BMI (Table 3) considered the extremes of the distribution more explicitly, albeit with very small sample size, and suggested that both overweight and underweight participants were at increased risk of mortality from cardiovascular disease, as well as all-cause mortality. Overweight and, particularly, obese participants were also at increased risk of subsequent death from cancers not related to smoking, but there was no evidence of increased mortality from this source in underweight participants. On the other hand, those who were overweight in early adulthood had a decreased risk of subsequently dying of smoking-related cancer, although the wide CI for this HR overlapped the null. Confidence intervals for the analysis by conventional categories of BMI were wide because of the low numbers of participants falling outside the recommended weight range.

Among the sensitivity analyses, neither the delayed follow-up, the additional adjustment nor the exclusion of those with missing smoking data had any substantial effect on the results (Supplementary Tables S7–S10). Restriction of the analysis to healthy non-smokers at age 20 reduced the sample size from 11,233 to 6,363 and the number of deaths from 2,438 to 1,108 (Table 2). The weakly negative association of BMI with smoking-related cancer in the full data was clearly positive when restricted to healthy non-smokers at age 20 and the positive association with cardiovascular disease mortality was amplified. Both restrictions, but particularly the restriction to non-smokers, appeared to be contributing to these changes (Supplementary Table S11). Restriction of the analysis of BMI in 2001 to those who were non-smokers at age 20 and never-smokers in 2001 amplified the positive association of BMI with cardiovascular disease mortality, although the confidence intervals overlapped considerably (Table 2). Restriction of the analysis to those with BMI between the 5^th^ and 95^th^ percentiles (i.e. 18.3 and 25.4 kg m^−2^; N = 10,118) slightly amplified the positive linear association with cardiovascular disease mortality and attenuated the positive linear association with mortality from cancers not related to smoking, although confidence intervals overlapped those from the original analysis (Supplementary Table S6). Most notably, the quadratic analysis of all-cause mortality no longer found evidence for a U-shaped curve; the association excluding these outliers could not be distinguished from linearity, and the fitted quadratic was weakly concave (inverse-U-shaped). There was also some evidence that the association of mortality from respiratory disease with BMI was concave after the exclusion of these outliers.

**Figure 3:**
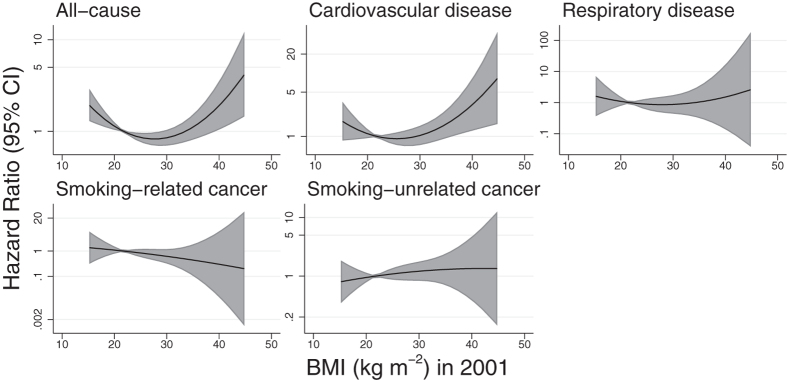
Fitted hazard ratios for all-cause and cause-specific mortality from the quadratic model of BMI in 2001. The midpoint of the recommended BMI range (18.5–25 kg m^−2^) is used as the reference point. Analyses were adjusted for sex, date of birth (cubic splines) and smoking behaviour in 2001.

In most cases, linear HR per 5 kg m^−2^ of BMI in 2001 (Table 2) were similar to those per 5 kg m^−2^ at age 20, and the confidence intervals overlapped in all cases. Quadratic models of mortality against BMI in 2001 (Table 2, Fig. 3) were suggestive of nonlinearity for both all-cause and cardiovascular disease mortality, with minimal mortality at a BMI falling in the overweight range (for all-cause mortality; 27.7 kg m^−2^ (95% CI: 25.5, 30.4). Plots by quartiles of BMI in 2001 (Supplementary Fig. S2) were suggestive of similarly U-shaped associations for all-cause and cardiovascular disease mortality, but with wide confidence intervals. The strongly negative linear association between respiratory disease mortality and BMI at age 20 was attenuated almost to the null when BMI in 2001 was used instead, with the suggestion of a U-shaped association with minimal mortality among those in the overweight range. Analysis of BMI in 2001 by categories (Table 3) found that for all causes of death except cancer not associated with smoking, overweight participants were at reduced risk of mortality compared to those in the recommended BMI range. Confidence intervals overlapped the null, but the negative association between all-cause mortality and overweight in 2001 was convincingly less than the positive association with overweight at age 20. Subjects had higher BMI on average in 2001 than at age 20, such that while obesity at age 20 was too scarce to analyse for most causes of death, in 2001 it was underweight that was particularly unusual and permitted only a limited analysis.

## Discussion

We found that participants with higher BMI in early adulthood were at greater risk of mortality from cardiovascular disease and cancers not related to smoking, but at reduced risk of mortality from (non-malignant) respiratory disease and perhaps from smoking-related cancers. With the possible exception of respiratory disease mortality, there was no evidence that these associations were nonlinear among those within the recommended BMI range who made up about 90% of the sample, but there was some evidence overall of a U-shaped association for cardiovascular disease, with the underweight and overweight both experiencing greater mortality than those in the recommended BMI range. All-cause mortality had a U-shaped association with BMI at age 20 or in 2001, but the optimum BMI shifted from the recommended range to the overweight range when BMI was recorded in 2001.

### Comparison with other studies

Since the data from this study were last analysed^12^ for nonlinearity, the mean follow-up has increased from 41 to 50 years and the number of deaths has more than doubled. For the causes of death previously analysed (all-cause, smoking-related cancer and smoking-unrelated cancer), HR per 5 kg m^−2^ of BMI were in the same direction as those calculated previously (except for smoking-related cancer, which was close to null in both analyses), but attenuated in the newer analysis. This might suggest that BMI has less influence on mortality rates in the very elderly than in the young or middle-aged, although the confidence intervals of all estimates overlapped considerably between the earlier and later studies, and there was no evidence in the current study that the proportional hazards assumption was violated.

A recent meta-analysis^4^ attracted considerable attention by concluding that all-cause mortality was lower among the overweight than among those of recommended BMI. Obese people had increased mortality and they did not report results for underweight. These results are similar to the results we found for BMI in 2001, but contrast with our results for BMI at age 20, in which overweight was associated with increased mortality. As well as differences due to the particular nature of our study population, it may be that the results of the meta-analysis were confounded by existing disease, since BMI in the meta-analysed studies, like our analysis of BMI in 2001, was measured with immediate follow-up.

Another large meta-analysis^1^ excluded the first five years of follow-up to limit the influence of reverse causality, and estimated HR per 5 kg m^−2^ separately for those with a BMI above and below 25 kg m^−2^, because mortality was minimised at a BMI between 22.5 and 25 kg m^−2^. Our quadratic analyses of BMI at age 20 suggested a slightly lower optimum BMI than this, while our analysis of BMI in 2001 suggested a considerably higher one. Extension of the lag in follow-up to 15 years in this meta-analysis attenuated the inverse association at lower BMI, suggesting that some confounding by reverse causality may have remained even when omitting the first five years of follow-up. They also found a much steeper inverse association at lower BMI for smokers than for non-smokers, suggesting some residual confounding by smoking behaviour (which was crudely adjusted for). Our finding that the linear association between smoking related cancer mortality and BMI at age 20 changed from negative to positive when restricted to healthy non-smokers (or just to non-smokers) is consistent with this finding. This result was repeated, albeit with reduced magnitude and very low precision, when BMI in 2001 was restricted to those who were non-smokers at age 20 and never-smokers in 2001.

A recent large Swedish study^13^ measured BMI in early adulthood and followed mortality into late middle age. Like the present study, they found that mortality was lowest among those whose BMI fell towards the lower end of the recommended range and that overweight was clearly associated with higher mortality.

### Strengths and limitations

The Glasgow Alumni Cohort differs from the modern British population in several important ways. Women are under-represented, and the results for women alone (Supplementary Table S5) did not have sufficient power do be interpreted with confidence. The students are from atypically high socioeconomic groups and the socioeconomic patterning of smoking, an important potential confounder of BMI and mortality, is reversed compared to modern populations^12^. Perhaps most importantly for the present study, only 7.7% of male and 7.4% of female participants were overweight or obese, compared with 35.2% of men and 36.9% of women aged 16–24 in the Scottish general population in 2011^14^.

BMI in this cohort, along with the potential confounding factors, was measured in early adulthood, long before the age at which there were substantial rates of mortality. This is both a strength and a weakness in this cohort. BMI later in life, integrating lifecourse exposures and closer to the age when most mortality occurs, might be of greater influence on mortality than BMI in early life although the strong conservation of BMI between early and late adulthood found here suggests that early-life BMI serves as a strong proxy for later-life BMI, as well as being a risk factor in its own right. Furthermore, because mortality rates are low in early adulthood the use of early adulthood BMI has a similar effect to the exclusion of follow-up for a period after BMI measurement (e.g. ref. 1) in reducing the risk of reverse causation.

Although BMI at age 20 was measured by a physician, BMI in 2001 was self-reported, which may have resulted in its underestimation, especially among the overweight^15^. Response levels were also low among those approached in 2001. Data were available to adjust to some extent for important potential confounders such as smoking and childhood SEP, but more comprehensive adjustment would have been desirable, in particular a direct measurement of physical activity (for which pulse rate is a crude proxy) or better quantification of smoking and SEP.

The characterisation of nonlinear associations is particularly demanding of data. While the present data were sufficient to identify simple quadratic nonlinearity in some associations, larger data sets such as those used in the cited meta-analyses would be needed to examine the shape of mortality-BMI associations in more detail or to test for more subtle nonlinearities with more precision. Such datasets, however, do not tend to have BMI measured in early adulthood and in middle age.

### What shape is the causal association between BMI and mortality?

A BMI of 18.5–25 kg m^−2^ is recommended to patients as a target for optimum health. Large conventional observational studies^4^ of the association between BMI and mortality, however, have led some to question whether parts of the BMI range currently considered to be overweight should be included within the recommended range, and indeed, whether the optimum BMI may lie within the overweight range. Such studies have also suggested a considerable adverse effect of even moderate underweight on survival. Critics of these analyses point out that confounding by smoking and by ill-health are both expected to associate lower BMI with elevated mortality, without there being a causal effect of BMI on mortality in this direction.

Our results suggested a U-shaped association between all-cause mortality and BMI, whether measured at age 20 or in 2001. However, the quadratic and categorical results for BMI in 2001 both suggested that overweight participants had lower mortality and that the optimum BMI fell within this range, while the equivalent results for BMI at age 20 showed higher mortality for the overweight, and an optimum BMI towards the lower end of the recommended range. This increase in the apparent optimum BMI with age has been found in a number of other studies^16^,^17^,^18^,^19^,^20^,^21^,^22^, some of which have suggested that the changing association is causal, indicating that the recommended BMI range should be made a function of age^16^,^19^,^20^. Alternative interpretations, however, include increasing effects of reverse causality or other confounding with age, a cumulative effect of lifecourse BMI and selective survival of the otherwise healthy among the overweight^19^ (but see ref. 22).

While the use of BMI in early adulthood has eliminated much of the risk of confounding by reverse causality, we cannot claim to have entirely eliminated this, or other sources of confounding such as smoking behaviour. For reverse causality to have confounded the results for BMI at age 20, the illness must already have been influencing subjects’ BMI at the time of their participation. While this is not implausible, it is unlikely to have been a major influence on BMI, given the time that elapsed before substantial numbers of subjects died and the minimal effect on HR of delaying follow-up by ten years.

Like most studies, we made a crude adjustment for smoking behaviour, distinguishing smokers from non-smokers at the time of participation. Such an adjustment can only give an approximate measure of a person’s lifetime exposure to tobacco, which can have a major influence both on BMI^23^,^24^,^25^ and on mortality^26^. Restriction of the analysis to healthy non-smokers at age 20 (Table 3) attenuated the excess mortality in the underweight and amplified the mortality in the overweight, suggesting that there was residual confounding by smoking, despite the short period in which smoking could have affected the BMI of these young men (but see ref. 27), and the limited association between BMI and smoking in our data (Table 1). Previous studies of older subjects^8^,^28^ have also found that the exclusion of ever-smokers and those with pre-existing disease shifts the apparent optimum BMI downwards into the recommended range.

## Conclusions

Our analysis of BMI in 2001, like some previous studies of BMI with immediate follow-up, found that overweight was associated with reduced mortality. In contrast, our analysis of BMI at age 20, like some studies of BMI with delayed follow-up, found that overweight was associated with increased mortality. While it is possible that the true optimum BMI for survival increases with age, increasing confounding by reverse causality is a more plausible interpretation. We suggest that increasing the recommended BMI range would not be justified by the evidence currently available.

Underweight at age 20 was associated with increased mortality later in life. While a harmful effect of extreme underweight is intuitively to be expected, the attenuation of this result when restricted to non-smokers suggests that confounding by smoking behaviour is at least partly responsible. Strong and changing associations of socioeconomic position with smoking, mortality and BMI make the interpretation of such associations challenging.

## Methods

### The Glasgow Alumni Cohort

The Glasgow Alumni Cohort consists of 11,755 male and 3,567 female students at the University of Glasgow who attended a health check between 1948 and 1968. All students were invited to health checks annually (we use the earliest available results for each student), and the cohort represents almost 50% of the student population over the study period. A physician took clinical measurements including height and weight and recorded information from the student including sociodemographic, behavioural and medical data. Further details of data collection are available elsewhere^29^. Approval to use the data was provided retrospectively by MREC Scotland. Deaths and emigration among participants were subsequently traced by the NHS central registers (NHSCR) in Edinburgh and Southport. In a follow-up study in 2000–2003, questionnaires asking health-related questions including current self-reported weight, height and smoking behaviour were sent to all traceable surviving participants (N = 11,422). For brevity, data collected during the university health checks are referred to as data “at age 20” and those collected in the later follow-up are referred to as data “in 2001”.

### Statistical Analysis

The cohort was followed up to 31^st^ December 2012. The NHSCR provided causes of death as ICD9 or ICD10 codes, which were converted into descriptive causes of death (Supplementary Table 1). Participants were excluded if they were older than 25 at first participation or lacked data on BMI at age 20 or (among those that died) on the cause of death (Fig. 1). For the analysis of BMI in 2001, data on BMI at this time were also required. Various health-related characteristics of the participants were summarised within quartiles of BMI at age 20 or in 2001, and linear or logistic regression, as appropriate, was used to quantify the association of each characteristic with BMI.

The association of all-cause and cause-specific mortality with BMI at age 20 was analysed for each sex in turn and for men and women combined, using Cox proportional hazards regression with age as the time axis. BMI was modelled in four different ways. First, BMI was included as a (log-) linear covariate giving HR per 5 kg m^−2^. Second, the logarithm of the HR was modelled as a quadratic function of BMI. The P-value from a Z-test of the second-order term was used to assess the evidence for quadratic nonlinearity. The vertex of fitted quadratic models was calculated to represent the BMI at which mortality was minimised (if the association was convex) or maximised (if the association was concave). Confidence intervals around the vertex were estimated using one million simulations of the coefficients of the quadratic model from their variance-covariance matrix because the probability distribution function of the vertex itself was not normally distributed^30^. Third, BMI was modelled in its conventional categories (Underweight, <18.5 kg m^−2^; Recommended, 18.5 − <25 kg m^−2^; Overweight, 25 − <30 kg m^−2^; Obese, ≥30 kg m^−2^), giving HR relative to the recommended BMI category. Finally, BMI was modelled as quartiles (which were plotted to assess the linearity of associations). In the analyses by categories of BMI, subjects were excluded from the analysis if they belonged to a BMI category in which there were no deaths. Follow-up was from the date of participation until the earliest of the subject’s death, emigration, or the 31^st^ December 2012. All models were adjusted for the subject’s date of birth as a cubic spline with knots at the 10^th^, 50^th^ and 90^th^ percentiles^31^ to allow for secular trends. Models were run with and without additional adjustment for smoking behaviour at age 20 (yes/no/missing). Combined analyses of men and women were also adjusted for sex.

The heterogeneity analysis from Stata’s metan command was used to test whether HR for BMI differed when the linear analyses were conducted separately on men and women (which would indicate a need to report separate analyses). To check the proportional hazards assumption, the Schoenfeld residuals for BMI from the linear model were tested for association with Ln(age). Five sensitivity analyses were conducted on the associations between mortality and BMI at age 20. First, to investigate the influence of outliers on the fitted quadratic associations, the analysis was repeated including only those subjects with a BMI between the 5^th^ and 95^th^ percentiles. Second, to reduce the risk of confounding by ill-health, analyses were repeated excluding the first ten years of each subject’s follow-up. Third, to avoid confounding by smoking status, the analysis was repeated using only healthy non-smokers at age 20, with healthy participants defined as those reporting no current complaints to the physician. Fourth, the analyses were repeated with additional adjustment for height (cubic spline with three knots), father’s occupation (four categories), birth order (first, second, third, or later), number of siblings (0, 1, 2, or more), pulse rate (linear) and age at menarche (≤11 years, 12–13 years, ≥14 years or male/missing). Finally, those participants missing data on smoking status at the time of BMI reporting were excluded, instead of missing data being treated as a different category.

At the follow-up study in 2001, participants included in the current analysis were between 49 and 78 years of age. Their BMI was therefore standardised using linear regression to its estimated value at the mean age of 63 before all further analyses. The association of all-cause and cause-specific mortality with BMI in 2001 was analysed using Cox regression models as described above, except that follow-up did not begin until the date of completion of the follow-up questionnaire and adjustment for smoking behaviour (current/former/never/missing) used data reported as part of the 2001 follow-up study. BMI in 2001 was analysed in linear and quadratic models, in conventional categories and as quartiles as described for BMI at age 20. As a sensitivity analysis, the analysis of BMI in 2001 was restricted to those who were non-smokers at age 20 and never-smokers in 2001. Additionally, BMI at age 20 was analysed as described previously, except that the inclusion criteria and follow-up period were those used in the analysis of BMI in 2001.

## Additional Information

**How to cite this article**: Carslake, D. *et al*. Being overweight in early adulthood is associated with increased mortality in middle age. *Sci. Rep.* **6**, 36046; doi: 10.1038/srep36046 (2016).

**Publisher’s note:** Springer Nature remains neutral with regard to jurisdictional claims in published maps and institutional affiliations.

## Supplementary Material

Supplementary Information

## Figures and Tables

**Table 1:** Baseline characteristics of participants by quartile of BMI at age 20.

Time, Measurement	Quartile of BMI at age 20^a^				Regression per 5 kg m^−2^		
	1^st^	2^nd^	3^rd^	4^th^	MD or OR	95% CI	N
aMeasured at age 20:							
bBMI (kg m^−2^)	18.9	20.7	22.0	24.4	5.00	(5.00, 5.00)	11,233
bAge (years)	19.6	19.7	19.8	20.0	0.34	(0.26, 0.41)	11,233
bHeight (cm)	172.1	172.6	172.6	171.3	−0.87	(−1.19, −0.55)	11,233
^b^Weight (kg)	56.0	61.7	65.6	71.8	14.0	(13.8, 14.3)	11,233
bPulse rate (bpm)	77.2	76.0	75.5	75.7	−0.9	(−1.3, −0.4)	10,876
bNumber of siblings	1.51	1.66	1.70	1.67	0.08	(0.02, 0.15)	11,230
cFemale (%)	26.6	22.3	20.0	23.1	0.84	(0.76, 0.93)	11,233
cFirst-born (%)	59.6	55.6	54.8	55.1	0.89	(0.82, 0.97)	11,233
cHigh paternal SEP (%)	56.5	58.0	56.7	57.6	1.03	(0.94, 1.12)	10,829
cSmokers (%)	28.8	29.9	27.4	29.2	1.00	(0.91, 1.10)	10,768
dMeasured in 2001:							
bBMI (kg m^−^2)	23.9	24.9	25.5	27.2	2.81	(2.62, 3.00)	4,841
bAge (years)	63.1	62.8	62.9	62.6	−0.46	(−0.89, −0.02)	4,841
bHeight (cm)	172.5	173.5	174.0	172.6	0.12	(−0.41, 0.66)	4,841
^b^Weight (kg)	71.3	75.2	77.4	81.2	8.5	(7.7, 9.2)	4,841
cSmokers (%)	7.5	9.1	10.5	10.6	1.29	(1.05, 1.58)	4,789
c^,^^e^Started smoking (%)	33.3	32.0	36.4	38.4	1.31	(1.12, 1.52)	3,424
c^,^^e^Stopped smoking (%)	86.8	79.2	80.7	78.5	0.74	(0.53, 1.01)	1,186

BMI: body mass index, CI: confidence interval, SEP: socioeconomic position, MD: mean difference, OR, odds ratio.

^a^Measured age 16–25.

^b^Interval-scale variables are summarised as means within each quartile of BMI and mean differences per 5 kg m^−2^ of BMI from unadjusted linear regression are presented.

^c^Binary variables are summarised as percentages within each quartile of BMI and odds ratios per 5 kg m^−2^ of BMI from unadjusted logistic regression are presented.

^d^Measured 2000–2002 and BMI standardised to age 63.

^e^The analysis of smoking uptake was restricted to non-smokers at age 20 and the analysis of smoking cessation was restricted to smokers at age 20.

**Table 2:** Linear hazard ratios for all-cause and cause-specific mortality per 5 kg m^−2^ of BMI.

Exposure, cause of death	Deaths	HR (95% CI)	P_quadratic_	Shape	BMI_vertex_ (95% CI)
*BMI at age 20, follow-up from age 20* (*N* = *11,233*):					
All cause	2,438	1.04 (0.95, 1.14)	0.021	convex	21.5 (16.7, 23.7)
Cardiovascular disease	804	1.13 (0.96, 1.32)	0.416	convex	18.6 (<11.8, >44.7)
Respiratory disease	173	0.61 (0.42, 0.87)	0.859	concave	<11.8 (<11.8, >44.7)
Smoking-related cancer	298	0.92 (0.71, 1.19)	0.880	concave	14.3 (<11.8, >44.7)
Cancer not smoking-related	647	1.22 (1.03, 1.45)	0.377	convex	16.6 (<11.8, >44.7)
*BMI in 2001, follow-up from 2001* (*N* = *4,841*):					
All cause	718	0.95 (0.85, 1.07)	0.001	convex	27.7 (25.5, 30.4)
Cardiovascular disease	231	1.10 (0.90, 1.35)	0.031	convex	25.5 (15.1, 29.4)
Respiratory disease	62	0.95 (0.64, 1.42)	0.544	convex	27.9 (<11.8, >44.7)
Smoking-related cancer	77	0.75 (0.52, 1.08)	0.913	concave	<11.8 (<11.8, >44.7)
Cancer not smoking-related	220	1.11 (0.91, 1.36)	0.840	concave	42.3 (<11.8, >44.7)
*BMI at age 20, follow-up from 2001* (*N* = *4,841*):					
All cause	718	1.06 (0.90, 1.26)	0.477	convex	20.4 (<11.8, >44.7)
Cardiovascular disease	231	1.15 (0.86, 1.54)	0.481	convex	19.7 (<11.8, >44.7)
Respiratory disease	62	0.76 (0.41, 1.41)	0.496	concave	20.0 (<11.8, 39.6)
Smoking-related cancer	77	0.84 (0.49, 1.43)	0.934	convex	36.5 (<11.8, >44.7)
Cancer not smoking-related	220	1.07 (0.79, 1.44)	0.952	concave	36.4 (<11.8, >44.7)
*BMI at age 20, follow-up from age 20, healthy non-smokers at age 20* (*N* = *6,363*):					
All cause	1,108	1.18 (1.04, 1.35)	0.051	convex	19.5 (<11.8, 26.1)
Cardiovascular disease	350	1.34 (1.06, 1.70)	0.315	convex	16.8 (<11.8, >44.7)
Respiratory disease	61	0.52 (0.28, 0.97)	0.906	concave	<11.8 (<11.8, >44.7)
Smoking-related cancer	104	1.64 (1.09, 2.46)	0.824	concave	>44.7 (<11.8, >44.7)
Cancer not smoking-related	354	1.21 (0.97, 1.52)	0.632	convex	14.8 (<11.8, >44.7)
*BMI in 2001, follow-up from 2001, never-smokers in 2001* (*N* = *2,226*):					
All cause	256	1.02 (0.84, 1.25)	0.091	convex	26.1 (14.5, 35.6)
Cardiovascular disease	74	1.26 (0.88, 1.80)	0.419	convex	21.9 (<11.8, >44.7)
Respiratory disease	19	0.74 (0.34, 1.63)	0.976	concave	<11.8 (<11.8, >44.7)
Smoking-related cancer	17	1.00 (0.46, 2.14)	0.778	concave	26.0 (<11.8, >44.7)
Cancer not smoking-related	99	1.18 (0.87, 1.58)	0.648	concave	33.1 (<11.8, >44.7)

BMI: body mass index, HR: hazard ratio, CI: confidence interval. BMI was measured at approximately age 20 and around 2001, when subjects were aged 49–78. Cox proportional hazards regression was used with age as the time axis. Models were adjusted for sex, date of birth (cubic splines) and smoking behaviour at the time of BMI reporting. Nonlinearity was assessed by adding BMI^2^ to the model and assessing its coefficient’s departure from the null (P_quadratic_). BMI_vertex_ was estimated as the BMI at which the tangent to the quadratic model was horizontal. In a convex quadratic curve (coefficient for BMI^2^ > 0), BMI_vertex_ estimates the BMI at which mortality is minimised. BMI_vertex_ values outside the observed range of BMI (11.8 to 44.7 kg m^−2^) indicate a monotonically increasing or decreasing association among the observed data and were abbreviated for ease of presentation.

**Table 3:** Hazard ratios for all-cause and cause-specific mortality, relative to recommended BMI (18.5 − <25 kg m^−2^), for underweight (<18.5 kg m^−2^), overweight (25 − <30 kg m^−2^) and obesity (≥30 kg m^−2^).

Cause of death	Deaths				HR (95% CI) relative to RW		
	UW	RW	OW	Ob	UW	OW	Ob
*BMI at age 20, follow-up from age 20* (*N* = *815 UW* + *9,708 RW* + *670 OW* + *40 Ob*):							
All cause	192	2077	160	9	1.21 (1.04, 1.40)	1.24 (1.05, 1.45)	1.51 (0.78, 2.90)
Cardiovascular disease	70	673	61	0	1.42 (1.11, 1.82)	1.51 (1.16, 1.96)	
Respiratory disease	15	153	5	0	1.35 (0.79, 2.30)	0.55 (0.22, 1.33)	
Smoking-related cancer	17	264	15	2	0.84 (0.51, 1.37)	0.92 (0.55, 1.55)	2.78 (0.69, 11.20)
Cancer not smoking-related	42	553	47	5	0.94 (0.69, 1.29)	1.33 (0.98, 1.79)	2.88 (1.19, 6.95)
*BMI in 2001, follow-up from 2001 (N* = *35 UW* + *2,393 RW* + *2,005 OW* + *408 Ob*):							
All cause	10	351	281	76	1.67 (0.89, 3.14)	0.86 (0.74, 1.01)	1.25 (0.98, 1.61)
Cardiovascular disease	3	104	92	32	1.70 (0.54, 5.37)	0.92 (0.69, 1.22)	1.77 (1.19, 2.64)
Respiratory disease	1	31	24	6	1.80 (0.24, 13.25)	0.82 (0.48, 1.40)	1.09 (0.45, 2.64)
Smoking-related cancer	1	41	28	7	1.24 (0.17, 9.03)	0.74 (0.46, 1.21)	0.93 (0.41, 2.07)
Cancer not smoking-related	2	102	94	22	1.18 (0.29, 4.80)	1.04 (0.78, 1.38)	1.30 (0.82, 2.07)
*BMI at age 20, follow-up from 2001* (*N* = *325 UW* + *4,209 RW* + *288 OW* + *19 Ob*):							
All cause	48	621	48	1	1.15 (0.86, 1.55)	1.34 (0.99, 1.79)	0.47 (0.07, 3.32)
Cardiovascular disease	17	193	21	0	1.35 (0.82, 2.23)	1.94 (1.24, 3.05)	
Respiratory disease	3	57	2	0	0.88 (0.28, 2.83)	0.66 (0.16, 2.70)	
Smoking-related cancer	5	67	5	0	1.06 (0.43, 2.64)	1.22 (0.49, 3.04)	
Cancer not smoking-related	14	192	14	0	1.03 (0.60, 1.78)	1.22 (0.71, 2.10)	
*BMI at age 20, follow-up from age 20, healthy non-smokers only* (*N* = *475 UW* + *5,474 RW* + *389 OW* + *25 Ob*):							
All cause	83	937	83	5	1.08 (0.87, 1.36)	1.49 (1.19, 1.87)	1.47 (0.61, 3.54)
Cardiovascular disease	31	288	31	0	1.38 (0.95, 2.01)	1.98 (1.37, 2.88)	
Respiratory disease	7	53	1	0	1.64 (0.74, 3.63)	0.33 (0.05, 2.38)	
Smoking-related cancer	3	92	7	2	0.40 (0.13, 1.26)	1.31 (0.61, 2.83)	6.35 (1.56, 25.79)
Cancer not smoking-related	22	302	28	2	0.85 (0.55, 1.31)	1.45 (0.99, 2.14)	1.75 (0.43, 7.02)
*BMI in 2001, follow-up from 2001, never-smokers in 2001* (*N* = *16 UW* + *1,170 RW* + *880 OW* + *160 Ob*):							
All cause	3	134	96	23	1.58 (0.50, 5.02)	0.89 (0.68, 1.16)	1.49 (0.96, 2.32)
Cardiovascular disease	1	38	25	10	2.21 (0.30, 16.38)	0.79 (0.48, 1.32)	2.43 (1.21, 4.89)
Respiratory disease	0	12	6	1		0.61 (0.23, 1.63)	0.73 (0.09, 5.66)
Smoking-related cancer	0	9	7	1		1.00 (0.37, 2.69)	0.96 (0.12, 7.67)
Cancer not smoking-related	1	47	43	8	1.35 (0.18, 9.95)	1.17 (0.77, 1.78)	1.39 (0.65, 2.94)

BMI: body mass index, UW: underweight, RW: recommended weight, OW: overweight, Ob: obese, HR: hazard ratio, CI: confidence interval. BMI was measured at approximately age 20 and around 2001, when subjects were aged 49–78. Cox proportional hazards regression was used with age as the time axis. All models were adjusted for sex, date of birth (cubic splines) and smoking behaviour at the time of BMI reporting (except where smokers were excluded). Where there were no deaths in a BMI category, participants falling into that category were removed from the analysis.
