# Supplementary material for: Being overweight in early adulthood is associated with increased mortality in middle age
Source: Sci Rep. 2016 Oct 26;6:36046. doi: 10.1038/srep36046 (PMC5080549; doi:10.1038/srep36046)

Supplementary material

Being overweight in early adulthood is associated with increased mortality in middle age

David Carslake[1,2*], Mona Jeffreys[3], George Davey Smith[1,2]

[1]MRC Integrative Epidemiology Unit at the University of Bristol, Oakfield House, Oakfield Grove, Bristol BS8 2BN, UK. [2]School of Social and Community Medicine, University of Bristol, Oakfield House, Oakfield Grove, Bristol BS8 2BN, UK. [3]School of Social and Community Medicine, University of Bristol, Canynge Hall, 39 Whatley Road, Bristol BS8 2PS, UK.
*Correspondence and requests for materials should be addressed to D. C. (email: David.Carslake@bristol.ac.uk).

Supplementary Table S1: Conversion of ICD codes into causes of death.
Supplementary Table S2. Baseline characteristics of participants by quartile of BMI in 2001.
Supplementary Table S3. Linear hazard ratios as in Table 2, but only adjusted for sex and date of birth (cubic splines).
Supplementary Table S4. Linear hazard ratios as in Table 2, but for men only.
Supplementary Table S5. Linear hazard ratios as in Table 2, but for women only.
Supplementary Table S6. Linear hazard ratios as in Table 2, but excluding BMIs outside the 5th - 95th percentiles.
Supplementary Table S7. Linear hazard ratios as in Table 2, but with follow-up beginning after a ten-year lag.
Supplementary Table S8. Hazard ratios by categories of BMI as in Table 3, but with follow-up beginning after a ten-year lag.
Supplementary Table S9. Linear hazard ratios as in Table 2, but with additional adjustment for father's social class, height (cubic splines), birth order, number of siblings, pulse rate and age at menarche.
Supplementary Table S10. Linear hazard ratios as in Table 2, but excluding those missing smoking data.
Supplementary Table S11. Linear hazard ratios as in Table 2, showing effects of restriction to (i) healthy non-smokers, (ii) healthy subjects and (iii) non-smokers, at age 20.

Supplementary Figure S1. Hazard ratios relative to the first quartile, for quartiles of BMI at age 20.
Supplementary Figure S2. Hazard ratios relative to the first quartile, for quartiles of BMI in 2001.
Supplementary Figure S3. Fitted quadratic hazard ratios as in Fig. 2, but excluding BMIs outside the 5th - 95th percentiles.
Supplementary Figure S4. Fitted quadratic hazard ratios as in Fig. 2, but with follow-up beginning after a ten-year lag.
Supplementary Figure S5. Fitted quadratic hazard ratios as in Fig. 2, but with additional adjustment for father's social class, height (cubic splines), birth order, number of siblings, pulse rate and age at menarche.
Supplementary Figure S6. Fitted quadratic hazard ratios as in Fig. 2, but excluding those missing smoking data.

Supplementary Table S1. Conversion of International Statistical Classification of Diseases and Related Health Problems (ICD) codes into causes of death. ICD 9 was used exclusively for deaths occurring until 31st December 2000 and ICD10 for deaths occurring from 1st November 2000, with a ten-month transition period between.

Cause of death	ICD 9	ICD 10
All cause	all	all
Cardiovascular disease	3900-4599	I000-I999; G450-G459
Respiratory disease	4600-5199	J000-J999
Smoking-related cancer	1400-1509; 1570-1579; 1600-1639; 1650-1659; 1880-1899	C000-C149; C250-C259; C300-C349; C390-C399; C640-C689
Cancer not smoking-related	1510-1569; 1580-1599; 1640-1649; 1700-1879; 1900-2089	C150-C249; C260-C269; C370-C389; C400-C639; C690-C979

Supplementary Table S2. Baseline characteristics of participants by quartile of BMI in 2001. BMI: body mass index, CI: confidence interval, SEP: socioeconomic position, MD: mean difference, OR, odds ratio. [a]Measured age 16-25. [b]Interval-scale variables are summarised as means within each quartile of BMI and mean differences per 5 kg m^{-2} of BMI from unadjusted linear regression are presented. [c]Binary variables are summarised as percentages within each quartile of BMI and odds ratios per 5 kg m^{-2} of BMI from unadjusted logistic regression are presented. [d]Measured 2000-2002 and BMI standardised to age 63. [e]The analysis of smoking uptake was restricted to non-smokers at age 20 and the analysis of smoking cessation was restricted to smokers at age 20.

	Quartile of BMI in 2001[a]				Regression per 5 kg m^{-2}		
Time, Measurement	1st	2nd	3rd	4th	MD or OR	95% CI	N
[a]Measured at age 20:							
[b]BMI (kg m^{-2})	20.6	21.1	21.6	22.7	1.27	(1.18, 1.35)	4,841
[b]Age (years)	19.6	19.6	19.8	19.7	0.05	(-0.02, 0.13)	4,841
[b]Height (cm)	170.8	172.4	173.0	171.8	0.22	(-0.12, 0.56)	4,841
[b]Weight (kg)	60.1	62.8	64.7	67.0	3.9	(3.5, 4.2)	4,841
[b]Pulse rate (bpm)	76.0	75.7	76.0	76.2	0.1	(-0.3, 0.5)	4,702
[b]Number of siblings	1.47	1.63	1.69	1.57	0.05	(-0.01, 0.11)	4,840
[c]Female (%)	38.2	22.9	17.9	23.4	0.74	(0.67, 0.82)	4,841
[c]First-born (%)	56.5	54.9	55.8	59.2	1.07	(0.98, 1.17)	4,841
[c]High paternal SEP (%)	61.5	58.7	57.9	54.8	0.88	(0.81, 0.96)	4,675
[c]Smokers (%)	23.2	25.0	26.3	28.4	1.09	(0.99, 1.20)	4,660
[d]Measured in 2001:							
[b]BMI (kg m^{-2})	21.5	24.1	26.0	29.8	5.00	(5.00, 5.00)	4,841
[b]Age (years)	62.3	62.9	63.4	62.8	0.11	(-0.18, 0.41)	4,841
[b]Height (cm)	172.1	173.6	174.1	172.8	-0.03	(-0.39, 0.33)	4,841
[b]Weight (kg)	63.9	72.8	79.0	89.1	14.7	(14.3, 15.0)	4,841
[c]Smokers (%)	11.7	8.4	8.4	9.2	0.85	(0.73, 0.99)	4,789
[c,e]Started smoking (%)	30.2	34.2	36.1	39.8	1.26	(1.14, 1.40)	3,424
[c,e]Stopped smoking (%)	72.1	84.1	85.2	82.1	1.49	(1.18, 1.89)	1,186

Supplementary Table S3. Linear hazard ratios for all-cause and cause-specific mortality per 5 kg m^{-2} of BMI. BMI: body mass index, HR: hazard ratio, CI: confidence interval. BMI was measured at approximately age 20 and around 2001, when subjects were aged 49-78. Cox proportional hazards regression was used with age as the time axis. Models were adjusted for sex and date of birth (cubic splines) only. Nonlinearity was assessed by adding BMI^2 to the model and assessing its coefficient's departure from the null ($P_{quadratic}$). BMI_{vertex} was estimated as the BMI at which the tangent to the quadratic model was horizontal. In a convex quadratic curve (coefficient for BMI^2>0), BMI_{vertex} estimates the BMI at which mortality is minimised. BMI_{vertex} values outside the observed range of BMI (11.8 to 44.7 kg m^{-2}) indicate a monotonically increasing or decreasing association among the observed data and were abbreviated for ease of presentation.

Exposure, cause of death	Deaths	HR (95% CI)	$P_{quadratic}$	Shape	BMI_{vertex} (95% CI)
BMI at age 20, follow-up from age 20 (N=11,233):					
All cause	2,438	1.04 (0.95, 1.14)	0.017	convex	21.5 (17.1, 23.6)
Cardiovascular disease	804	1.13 (0.96, 1.32)	0.377	convex	18.8 (<11.8, >44.7)
Respiratory disease	173	0.60 (0.42, 0.87)	0.922	concave	<11.8 (<11.8, >44.7)
Smoking-related cancer	298	0.91 (0.70, 1.19)	0.932	concave	<11.8 (<11.8, >44.7)
Cancer not smoking-related	647	1.22 (1.03, 1.45)	0.378	convex	16.6 (<11.8, >44.7)
BMI in 2001, follow-up from 2001 (N=4,841):					
All cause	718	0.94 (0.84, 1.06)	0.000	convex	27.8 (25.9, 30.2)
Cardiovascular disease	231	1.10 (0.90, 1.34)	0.017	convex	25.9 (18.0, 29.0)
Respiratory disease	62	0.93 (0.63, 1.39)	0.507	convex	28.2 (<11.8, >44.7)
Smoking-related cancer	77	0.75 (0.52, 1.08)	0.933	convex	>44.7 (<11.8, >44.7)
Cancer not smoking-related	220	1.09 (0.89, 1.33)	0.890	concave	>44.7 (<11.8, >44.7)
BMI at age 20, follow-up from 2001 (N=4,841):					
All cause	718	1.07 (0.90, 1.26)	0.477	convex	20.1 (<11.8, >44.7)
Cardiovascular disease	231	1.16 (0.86, 1.56)	0.476	convex	19.5 (<11.8, >44.7)
Respiratory disease	62	0.77 (0.42, 1.44)	0.502	concave	20.1 (<11.8, 39.4)
Smoking-related cancer	77	0.85 (0.50, 1.44)	0.954	convex	41.9 (<11.8, >44.7)
Cancer not smoking-related	220	1.07 (0.79, 1.44)	0.956	concave	37.5 (<11.8, >44.7)
BMI at age 20, follow-up from age 20, healthy non-smokers at age 20 (N=6,363):					
All cause	1,108	1.18 (1.04, 1.35)	0.051	convex	19.5 (<11.8, 26.2)
Cardiovascular disease	350	1.34 (1.06, 1.70)	0.315	convex	16.8 (<11.8, >44.7)
Respiratory disease	61	0.52 (0.28, 0.97)	0.906	concave	<11.8 (<11.8, >44.7)
Smoking-related cancer	104	1.64 (1.09, 2.46)	0.824	concave	>44.7 (<11.8, >44.7)
Cancer not smoking-related	354	1.21 (0.97, 1.52)	0.632	convex	14.8 (<11.8, >44.7)
BMI in 2001, follow-up from 2001, never-smokers in 2001 (N=2,226):					
All cause	256	1.02 (0.84, 1.25)	0.091	convex	26.1 (14.5, 35.7)
Cardiovascular disease	74	1.26 (0.88, 1.80)	0.419	convex	21.9 (<11.8, >44.7)
Respiratory disease	19	0.74 (0.34, 1.63)	0.976	concave	<11.8 (<11.8, >44.7)
Smoking-related cancer	17	1.00 (0.46, 2.14)	0.778	concave	26.0 (<11.8, >44.7)
Cancer not smoking-related	99	1.18 (0.87, 1.58)	0.648	concave	33.1 (<11.8, >44.7)

Supplementary Table S4. Linear hazard ratios for all-cause and cause-specific mortality <u>in men only,</u> per 5 kg m^{-2} of BMI. BMI: body mass index, HR: hazard ratio, CI: confidence interval. BMI was measured at approximately age 20 and around 2001, when subjects were aged 49-78. Cox proportional hazards regression was used with age as the time axis. Models were adjusted for sex, date of birth (cubic splines) and smoking behaviour at the time of BMI reporting. Nonlinearity was assessed by adding BMI^2 to the model and assessing its coefficient's departure from the null ($P_{quadratic}$). BMI_{vertex} was estimated as the BMI at which the tangent to the quadratic model was horizontal. In a convex quadratic curve (coefficient for BMI^2>0), BMI_{vertex} estimates the BMI at which mortality is minimised. BMI_{vertex} values outside the observed range of BMI (11.8 to 44.7 kg m^{-2}) indicate a monotonically increasing or decreasing association among the observed data and were abbreviated for ease of presentation.

Exposure, cause of death	Deaths	HR (95% CI)	$P_{quadratic}$	Shape	BMI_{vertex} (95% CI)
BMI at age 20, follow-up from age 20 (N=8,648):					
All cause	2,040	1.03 (0.93, 1.14)	0.022	convex	21.8 (17.7, 24.3)
Cardiovascular disease	728	1.15 (0.97, 1.35)	0.435	convex	18.2 (<11.8, >44.7)
Respiratory disease	147	0.62 (0.41, 0.93)	0.982	concave	<11.8 (<11.8, >44.7)
Smoking-related cancer	246	0.90 (0.67, 1.21)	0.837	convex	28.4 (<11.8, >44.7)
Cancer not smoking-related	494	1.18 (0.96, 1.44)	0.127	convex	20.1 (<11.8, 38.8)
BMI in 2001, follow-up from 2001 (N=3,602):					
All cause	603	1.01 (0.88, 1.15)	0.001	convex	26.8 (24.1, 29.0)
Cardiovascular disease	206	1.17 (0.94, 1.46)	0.016	convex	25.1 (15.2, 28.1)
Respiratory disease	53	0.94 (0.59, 1.48)	0.195	convex	27.5 (<11.8, >44.7)
Smoking-related cancer	61	0.77 (0.50, 1.19)	0.734	concave	17.7 (<11.8, >44.7)
Cancer not smoking-related	176	1.25 (0.99, 1.58)	0.506	concave	34.7 (<11.8, >44.7)
BMI at age 20, follow-up from 2001 (N=3,602):					
All cause	603	1.07 (0.88, 1.29)	0.899	concave	32.6 (<11.8, >44.7)
Cardiovascular disease	206	1.26 (0.92, 1.72)	0.542	convex	17.5 (<11.8, >44.7)
Respiratory disease	53	0.74 (0.38, 1.47)	0.383	concave	20.5 (<11.8, 35.0)
Smoking-related cancer	61	0.68 (0.36, 1.27)	0.754	concave	15.8 (<11.8, >44.7)
Cancer not smoking-related	176	0.99 (0.70, 1.41)	0.805	concave	21.9 (<11.8, >44.7)
BMI at age 20, follow-up from age 20, healthy non-smokers at age 20 (N=4,732):					
All cause	877	1.22 (1.05, 1.42)	0.368	convex	16.2 (<11.8, >44.7)
Cardiovascular disease	310	1.43 (1.11, 1.83)	0.472	convex	13.7 (<11.8, >44.7)
Respiratory disease	45	0.58 (0.28, 1.22)	0.590	concave	17.7 (<11.8, >44.7)
Smoking-related cancer	76	1.90 (1.18, 3.08)	0.761	convex	<11.8 (<11.8, >44.7)
Cancer not smoking-related	254	1.20 (0.91, 1.59)	0.945	convex	<11.8 (<11.8, >44.7)
BMI in 2001, follow-up from 2001, never-smokers in 2001 (N=1,513):					
All cause	207	1.10 (0.87, 1.39)	0.069	convex	25.3 (<11.8, 33.4)
Cardiovascular disease	64	1.30 (0.86, 1.96)	0.228	convex	23.4 (<11.8, >44.7)
Respiratory disease	16	0.69 (0.27, 1.74)	0.495	convex	29.5 (<11.8, >44.7)
Smoking-related cancer	13	1.40 (0.56, 3.46)	0.767	concave	32.5 (<11.8, >44.7)
Cancer not smoking-related	74	1.24 (0.84, 1.82)	0.453	concave	29.6 (<11.8, >44.7)

Supplementary Table S5. Linear hazard ratios for all-cause and cause-specific mortality in women only, per 5 kg m^{-2} of BMI. BMI: body mass index, HR: hazard ratio, CI: confidence interval. BMI was measured at approximately age 20 and around 2001, when subjects were aged 49-78. Cox proportional hazards regression was used with age as the time axis. Models were adjusted for sex, date of birth (cubic splines) and smoking behaviour at the time of BMI reporting. Nonlinearity was assessed by adding BMI^2 to the model and assessing its coefficient's departure from the null ($P_{quadratic}$). BMI_{vertex} was estimated as the BMI at which the tangent to the quadratic model was horizontal. In a convex quadratic curve (coefficient for BMI^2>0), BMI_{vertex} estimates the BMI at which mortality is minimised. BMI_{vertex} values outside the observed range of BMI (11.8 to 44.7 kg m^{-2}) indicate a monotonically increasing or decreasing association among the observed data and were abbreviated for ease of presentation.

Exposure, cause of death	Deaths	HR (95% CI)	$P_{quadratic}$	Shape	BMI_{vertex} (95% CI)
BMI at age 20, follow-up from age 20 (N=2,585):					
All cause	398	1.10 (0.90, 1.34)	0.477	convex	19.3 (<11.8, >44.7)
Cardiovascular disease	76	0.99 (0.63, 1.58)	0.908	convex	22.4 (<11.8, >44.7)
Respiratory disease	26	0.56 (0.24, 1.28)	0.659	concave	16.1 (<11.8, >44.7)
Smoking-related cancer	52	1.00 (0.57, 1.74)	0.565	concave	22.0 (<11.8, 41.1)
Cancer not smoking-related	153	1.36 (0.99, 1.85)	0.543	concave	28.7 (<11.8, >44.7)
BMI in 2001, follow-up from 2001 (N=1,239):					
All cause	115	0.81 (0.63, 1.04)	0.336	convex	32.4 (<11.8, >44.7)
Cardiovascular disease	25	0.80 (0.47, 1.37)	0.691	concave	20.8 (<11.8, >44.7)
Respiratory disease	9	1.02 (0.45, 2.32)	0.277	concave	26.0 (11.8, 40.2)
Smoking-related cancer	16	0.70 (0.34, 1.46)	0.635	convex	32.0 (<11.8, >44.7)
Cancer not smoking-related	44	0.83 (0.56, 1.24)	0.559	convex	31.7 (<11.8, >44.7)
BMI at age 20, follow-up from 2001 (N=1,239):					
All cause	115	1.03 (0.71, 1.50)	0.074	convex	22.3 (15.1, 28.1)
Cardiovascular disease	25	0.58 (0.24, 1.43)	0.890	concave	<11.8 (<11.8, >44.7)
Respiratory disease	9	0.86 (0.19, 3.84)	0.799	convex	23.6 (<11.8, >44.7)
Smoking-related cancer	16	1.47 (0.56, 3.83)	0.785	convex	16.7 (<11.8, >44.7)
Cancer not smoking-related	44	1.30 (0.74, 2.30)	0.893	convex	<11.8 (<11.8, >44.7)
BMI at age 20, follow-up from age 20, healthy non-smokers at age 20 (N=1,631):					
All cause	231	1.10 (0.84, 1.43)	0.016	convex	21.8 (16.6, 24.2)
Cardiovascular disease	40	0.92 (0.48, 1.76)	0.438	convex	23.1 (<11.8, 44.4)
Respiratory disease	16	0.40 (0.13, 1.28)	0.614	convex	28.3 (<11.8, >44.7)
Smoking-related cancer	28	1.20 (0.57, 2.53)	0.337	concave	22.9 (<11.8, 37.1)
Cancer not smoking-related	100	1.28 (0.87, 1.88)	0.467	convex	18.5 (<11.8, >44.7)
BMI in 2001, follow-up from 2001, never-smokers in 2001 (N=713):					
All cause	49	0.85 (0.59, 1.23)	0.700	convex	33.7 (<11.8, >44.7)
Cardiovascular disease	10	1.13 (0.54, 2.38)	0.653	concave	28.7 (<11.8, >44.7)
Respiratory disease	3	0.00 (0.00, 0.00)	0.000	concave	<11.8 (<11.8, <11.8)
Smoking-related cancer	4	0.00 (0.00, 0.00)	0.000	concave	<11.8 (<11.8, <11.8)
Cancer not smoking-related	25	1.03 (0.64, 1.66)	0.875	convex	24.7 (<11.8, >44.7)

Supplementary Table S6. Linear hazard ratios for all-cause and cause-specific mortality per 5 kg m^{-2} of BMI, <u>including only those with BMI between the 5th and 95th percentiles</u>. BMI: body mass index, HR: hazard ratio, CI: confidence interval. BMI was measured at approximately age 20 and around 2001, when subjects were aged 49-78. Cox proportional hazards regression was used with age as the time axis. Models were adjusted for sex, date of birth (cubic splines) and smoking behaviour at the time of BMI reporting. Nonlinearity was assessed by adding BMI^2 to the model and assessing its coefficient's departure from the null ($P_{quadratic}$). BMI_{vertex} was estimated as the BMI at which the tangent to the quadratic model was horizontal. In a convex quadratic curve (coefficient for BMI^2>0), BMI_{vertex} estimates the BMI at which mortality is minimised. BMI_{vertex} values outside the observed range of BMI (11.8 to 44.7 kg m^{-2}) indicate a monotonically increasing or decreasing association among the observed data and were abbreviated for ease of presentation.

Exposure, cause of death	Deaths	HR (95% CI)	$P_{quadratic}$	Shape	BMI_{vertex} (95% CI)
BMI at age 20, follow-up from age 20 (N=10,118):					
All cause	2,167	1.03 (0.91, 1.17)	0.295	concave	22.0 (14.4, 29.5)
Cardiovascular disease	708	1.26 (1.01, 1.58)	0.638	concave	25.8 (<11.8, >44.7)
Respiratory disease	155	0.64 (0.40, 1.04)	0.048	concave	20.6 (16.5, 22.1)
Smoking-related cancer	272	0.83 (0.58, 1.19)	0.233	concave	20.7 (12.3, 29.0)
Cancer not smoking-related	575	1.09 (0.86, 1.39)	0.825	convex	18.6 (<11.8, 36.0)
BMI in 2001, follow-up from 2001 (N=2,693):					
All cause	386	0.61 (0.45, 0.83)	0.876	concave	<11.8 (<11.8, >44.7)
Cardiovascular disease	122	0.93 (0.53, 1.62)	0.863	convex	24.2 (<11.8, 35.6)
Respiratory disease	34	0.55 (0.20, 1.51)	0.469	concave	21.0 (<11.8, 36.7)
Smoking-related cancer	43	0.38 (0.16, 0.90)	0.337	concave	20.2 (<11.8, 41.7)
Cancer not smoking-related	111	1.18 (0.65, 2.13)	0.679	concave	23.9 (<11.8, 35.6)
BMI at age 20, follow-up from 2001 (N=4,359):					
All cause	641	1.09 (0.86, 1.38)	0.491	concave	22.6 (<11.8, 33.6)
Cardiovascular disease	199	1.10 (0.72, 1.67)	0.107	concave	21.8 (18.6, 25.7)
Respiratory disease	58	0.64 (0.29, 1.43)	0.125	concave	20.9 (15.8, 25.0)
Smoking-related cancer	70	0.63 (0.31, 1.30)	0.451	convex	23.1 (<11.8, 37.8)
Cancer not smoking-related	196	1.21 (0.79, 1.85)	0.826	convex	17.9 (<11.8, 37.3)
BMI at age 20, follow-up from age 20, healthy non-smokers at age 20 (N=5,728):					
All cause	978	1.16 (0.96, 1.41)	0.793	convex	15.8 (<11.8, >44.7)
Cardiovascular disease	303	1.46 (1.04, 2.05)	0.872	convex	<11.8 (<11.8, >44.7)
Respiratory disease	53	0.76 (0.33, 1.75)	0.201	concave	21.0 (15.2, 26.7)
Smoking-related cancer	95	1.42 (0.78, 2.59)	0.640	concave	24.0 (<11.8, 39.1)
Cancer not smoking-related	313	1.11 (0.80, 1.55)	0.943	concave	30.1 (<11.8, 36.2)
BMI in 2001, follow-up from 2001, never-smokers in 2001 (N=1,194):					
All cause	135	0.78 (0.45, 1.34)	0.965	convex	40.9 (<11.8, 38.0)
Cardiovascular disease	38	1.27 (0.42, 3.86)	0.144	concave	22.8 (19.4, 26.3)
Respiratory disease	12	0.84 (0.13, 5.35)	0.862	concave	21.4 (<11.8, 33.5)
Smoking-related cancer	9	0.75 (0.10, 5.52)	0.159	concave	22.2 (18.3, 25.8)
Cancer not smoking-related	48	1.90 (0.70, 5.13)	0.792	convex	18.1 (<11.8, 41.4)

Supplementary Table S7. Linear hazard ratios for all-cause and cause-specific mortality per 5 kg m^{-2} of BMI, <u>with follow-up beginning after a ten-year lag</u>. BMI: body mass index, HR: hazard ratio, CI: confidence interval. BMI was measured at approximately age 20 and around 2001, when subjects were aged 49-78. Cox proportional hazards regression was used with age as the time axis. Models were adjusted for sex, date of birth (cubic splines) and smoking behaviour at the time of BMI reporting. Nonlinearity was assessed by adding BMI^2 to the model and assessing its coefficient's departure from the null ($P_{quadratic}$). BMI_{vertex} was estimated as the BMI at which the tangent to the quadratic model was horizontal. In a convex quadratic curve (coefficient for BMI^2>0), BMI_{vertex} estimates the BMI at which mortality is minimised. BMI_{vertex} values outside the observed range of BMI (11.8 to 44.7 kg m^{-2}) indicate a monotonically increasing or decreasing association among the observed data and were abbreviated for ease of presentation.

Exposure, cause of death	Deaths	HR (95% CI)	$P_{quadratic}$	Shape	BMI_{vertex} (95% CI)
BMI at age 20, follow-up from age 20 (N=10,963):					
All cause	2,420	1.04 (0.95, 1.14)	0.021	convex	21.4 (16.5, 23.7)
Cardiovascular disease	800	1.13 (0.97, 1.33)	0.402	convex	18.6 (<11.8, >44.7)
Respiratory disease	172	0.61 (0.42, 0.88)	0.877	concave	<11.8 (<11.8, >44.7)
Smoking-related cancer	298	0.92 (0.71, 1.19)	0.880	concave	14.3 (<11.8, >44.7)
Cancer not smoking-related	644	1.21 (1.02, 1.43)	0.411	convex	16.5 (<11.8, >44.7)
BMI in 2001, follow-up from 2001 (N=4,841):					
All cause	718	0.95 (0.85, 1.07)	0.001	convex	27.7 (25.5, 30.4)
Cardiovascular disease	231	1.10 (0.91, 1.35)	0.031	convex	25.5 (15.1, 29.4)
Respiratory disease	62	0.95 (0.64, 1.42)	0.544	convex	27.9 (<11.8, >44.7)
Smoking-related cancer	77	0.75 (0.52, 1.08)	0.913	concave	<11.8 (<11.8, >44.7)
Cancer not smoking-related	220	1.11 (0.91, 1.36)	0.840	concave	42.3 (<11.8, >44.7)
BMI at age 20, follow-up from 2001 (N=4,841):					
All cause	718	1.06 (0.90, 1.26)	0.477	convex	20.4 (<11.8, >44.7)
Cardiovascular disease	231	1.15 (0.86, 1.54)	0.481	convex	19.7 (<11.8, >44.7)
Respiratory disease	62	0.76 (0.41, 1.41)	0.496	concave	20.0 (<11.8, 39.6)
Smoking-related cancer	77	0.84 (0.49, 1.43)	0.934	convex	36.5 (<11.8, >44.7)
Cancer not smoking-related	220	1.07 (0.79, 1.44)	0.952	concave	36.5 (<11.8, >44.7)
BMI at age 20, follow-up from age 20, healthy non-smokers at age 20 (N=6,214):					
All cause	1,099	1.18 (1.04, 1.35)	0.045	convex	19.6 (<11.8, 22.8)
Cardiovascular disease	348	1.36 (1.08, 1.72)	0.335	convex	16.4 (<11.8, >44.7)
Respiratory disease	61	0.52 (0.28, 0.97)	0.906	concave	<11.8 (<11.8, >44.7)
Smoking-related cancer	104	1.64 (1.09, 2.46)	0.824	concave	>44.7 (<11.8, >44.7)
Cancer not smoking-related	352	1.21 (0.96, 1.52)	0.599	convex	15.5 (<11.8, >44.7)
BMI in 2001, follow-up from 2001, never-smokers in 2001 (N=2,226):					
All cause	256	1.02 (0.84, 1.25)	0.091	convex	26.1 (14.5, 35.6)
Cardiovascular disease	74	1.26 (0.88, 1.80)	0.419	convex	21.9 (<11.8, >44.7)
Respiratory disease	19	0.74 (0.34, 1.63)	0.976	concave	<11.8 (<11.8, >44.7)
Smoking-related cancer	17	1.00 (0.46, 2.14)	0.778	concave	26.0 (<11.8, >44.7)
Cancer not smoking-related	99	1.18 (0.87, 1.58)	0.648	concave	33.1 (<11.8, >44.7)

Supplementary Table S8. Hazard ratios for all-cause and cause-specific mortality, relative to recommended BMI (18.5-<25 kg m^{-2}), for underweight (<18.5 kg m^{-2}), overweight (25-<30 kg m^{-2}) and obesity (≥30 kg m^{-2}), <u>with follow-up beginning after a ten-year lag</u>. BMI: body mass index, DOB: date of birth, UW: underweight, RW: recommended weight, OW: overweight, Ob: obese, HR: hazard ratio, CI: confidence interval. BMI was measured at approximately age 20 and around 2001, when subjects were aged 49-78. Cox proportional hazards regression was used with age as the time axis. All models were adjusted for sex, cubic splines of DOB and smoking behaviour at the time of BMI reporting (except where smokers were excluded). Where there were no deaths in a BMI category, participants falling into that category were removed from the analysis.

	Deaths				HR (95% CI) relative to RW		
Cause of death	UW	RW	OW	Ob	UW	OW	Ob
BMI at age 20, follow-up from age 20 (N = 798 UW + 9,466 RW + 660 OW + 39 Ob):							
All cause	191	2061	159	9	1.21 (1.05, 1.41)	1.24 (1.06, 1.46)	1.53 (0.79, 2.94)
Cardiovascular disease	70	669	61	0	1.43 (1.12, 1.83)	1.51 (1.16, 1.97)	
Respiratory disease	15	152	5	0	1.36 (0.80, 2.31)	0.55 (0.23, 1.34)	
Smoking-related cancer	17	264	15	2	0.84 (0.51, 1.37)	0.92 (0.55, 1.55)	2.78 (0.69, 11.20)
Cancer not smoking-related	42	551	46	5	0.94 (0.69, 1.29)	1.30 (0.97, 1.76)	2.90 (1.20, 7.01)
BMI in 2001, follow-up from 2001 (N = 35 UW + 2,393 RW + 2,005 OW + 408 Ob):							
All cause	10	351	281	76	1.67 (0.89, 3.14)	0.86 (0.74, 1.01)	1.25 (0.98, 1.61)
Cardiovascular disease	3	104	92	32	1.70 (0.54, 5.37)	0.92 (0.69, 1.22)	1.77 (1.19, 2.64)
Respiratory disease	1	31	24	6	1.80 (0.24, 13.25)	0.82 (0.48, 1.40)	1.09 (0.45, 2.64)
Smoking-related cancer	1	41	28	7	1.24 (0.17, 9.03)	0.74 (0.46, 1.21)	0.93 (0.41, 2.07)
Cancer not smoking-related	2	102	94	22	1.18 (0.29, 4.80)	1.04 (0.78, 1.38)	1.30 (0.82, 2.07)
BMI at age 20, follow-up from 2001 (N = 325 UW + 4,209 RW + 288 OW + 19 Ob):							
All cause	48	621	48	1	1.15 (0.86, 1.55)	1.34 (0.99, 1.79)	0.47 (0.07, 3.32)
Cardiovascular disease	17	193	21	0	1.35 (0.82, 2.23)	1.94 (1.24, 3.05)	
Respiratory disease	3	57	2	0	0.88 (0.28, 2.83)	0.66 (0.16, 2.69)	
Smoking-related cancer	5	67	5	0	1.06 (0.43, 2.64)	1.22 (0.49, 3.04)	
Cancer not smoking-related	14	192	14	0	1.03 (0.60, 1.78)	1.22 (0.71, 2.10)	
BMI at age 20, follow-up from age 20, healthy non-smokers only (N = 466 UW + 5,338 RW + 386 OW + 24 Ob):							
All cause	83	928	83	5	1.10 (0.87, 1.37)	1.51 (1.20, 1.89)	1.49 (0.62, 3.59)
Cardiovascular disease	31	286	31	0	1.39 (0.96, 2.02)	2.00 (1.38, 2.90)	
Respiratory disease	7	53	1	0	1.64 (0.74, 3.63)	0.33 (0.05, 2.38)	
Smoking-related cancer	3	92	7	2	0.40 (0.13, 1.26)	1.31 (0.61, 2.83)	6.35 (1.56, 25.79)
Cancer not smoking-related	22	300	28	2	0.85 (0.55, 1.32)	1.46 (0.99, 2.16)	1.76 (0.44, 7.08)
BMI in 2001, follow-up from 2001, never-smokers in 2001 (N = 16 UW + 1,170 RW + 880 OW + 160 Ob):							
All cause	3	134	96	23	1.58 (0.50, 5.02)	0.89 (0.68, 1.16)	1.49 (0.96, 2.32)
Cardiovascular disease	1	38	25	10	2.21 (0.30, 16.39)	0.79 (0.48, 1.32)	2.43 (1.21, 4.89)
Respiratory disease	0	12	6	1		0.61 (0.23, 1.63)	0.73 (0.09, 5.66)
Smoking-related cancer	0	9	7	1		1.00 (0.37, 2.69)	0.96 (0.12, 7.67)
Cancer not smoking-related	1	47	43	8	1.35 (0.18, 9.95)	1.17 (0.77, 1.78)	1.39 (0.65, 2.94)

Supplementary Table S9. Linear hazard ratios for all-cause and cause-specific mortality per 5 kg m^{-2} of BMI, with additional adjustment. BMI: body mass index, HR: hazard ratio, CI: confidence interval. BMI was measured at approximately age 20 and around 2001, when subjects were aged 49-78. Cox proportional hazards regression was used with age as the time axis. Models were adjusted for sex, date of birth (cubic splines), smoking behaviour at the time of BMI reporting, father's social class, height (cubic splines), birth order, number of siblings, pulse rate and age at menarche. Nonlinearity was assessed by adding BMI^2 to the model and assessing its coefficient's departure from the null ($P_{quadratic}$). BMI_{vertex} was estimated as the BMI at which the tangent to the quadratic model was horizontal. In a convex quadratic curve (coefficient for BMI^2>0), BMI_{vertex} estimates the BMI at which mortality is minimised. BMI_{vertex} values outside the observed range of BMI (11.8 to 44.7 kg m^{-2}) indicate a monotonically increasing or decreasing association among the observed data and were abbreviated for ease of presentation.

Exposure, cause of death	Deaths	HR (95% CI)	$P_{quadratic}$	Shape	BMI_{vertex} (95% CI)
BMI at age 20, follow-up from age 20 (N=10,876):					
All cause	2,397	1.03 (0.94, 1.13)	0.059	convex	21.6 (14.7, 26.0)
Cardiovascular disease	793	1.09 (0.92, 1.27)	0.432	convex	19.7 (<11.8, >44.7)
Respiratory disease	171	0.61 (0.42, 0.89)	0.785	concave	<11.8 (<11.8, >44.7)
Smoking-related cancer	294	0.94 (0.72, 1.23)	0.874	concave	17.1 (<11.8, >44.7)
Cancer not smoking-related	636	1.23 (1.03, 1.46)	0.654	convex	<11.8 (<11.8, >44.7)
BMI in 2001, follow-up from 2001 (N=4,702):					
All cause	713	0.95 (0.85, 1.07)	0.001	convex	27.6 (25.5, 30.4)
Cardiovascular disease	231	1.10 (0.90, 1.34)	0.037	convex	25.6 (14.5, 30.1)
Respiratory disease	61	0.90 (0.60, 1.36)	0.663	convex	30.0 (<11.8, >44.7)
Smoking-related cancer	77	0.73 (0.51, 1.05)	0.913	concave	<11.8 (<11.8, >44.7)
Cancer not smoking-related	218	1.14 (0.93, 1.39)	0.900	concave	>44.7 (<11.8, >44.7)
BMI at age 20, follow-up from 2001 (N=4,702):					
All cause	713	1.08 (0.91, 1.28)	0.545	convex	19.3 (<11.8, >44.7)
Cardiovascular disease	231	1.11 (0.82, 1.49)	0.605	convex	19.7 (<11.8, >44.7)
Respiratory disease	61	0.79 (0.43, 1.48)	0.446	concave	20.5 (<11.8, 36.9)
Smoking-related cancer	77	0.87 (0.51, 1.48)	0.908	convex	30.3 (<11.8, >44.7)
Cancer not smoking-related	218	1.15 (0.85, 1.55)	0.806	concave	29.4 (<11.8, >44.7)
BMI at age 20, follow-up from age 20, healthy non-smokers at age 20 (N=6,146):					
All cause	1,089	1.17 (1.02, 1.34)	0.052	convex	19.7 (<11.8, 26.1)
Cardiovascular disease	346	1.30 (1.02, 1.65)	0.264	convex	18.1 (<11.8, >44.7)
Respiratory disease	61	0.51 (0.27, 0.96)	0.934	concave	<11.8 (<11.8, >44.7)
Smoking-related cancer	104	1.61 (1.07, 2.42)	0.789	concave	40.9 (<11.8, >44.7)
Cancer not smoking-related	348	1.24 (0.98, 1.56)	0.675	convex	12.9 (<11.8, >44.7)
BMI in 2001, follow-up from 2001, never-smokers in 2001 (N=2,162):					
All cause	255	1.03 (0.84, 1.26)	0.140	convex	26.0 (<11.8, 39.6)
Cardiovascular disease	74	1.21 (0.84, 1.73)	0.506	convex	21.9 (<11.8, >44.7)
Respiratory disease	19	0.71 (0.32, 1.57)	0.891	concave	<11.8 (<11.8, >44.7)
Smoking-related cancer	17	1.06 (0.48, 2.35)	0.749	concave	27.3 (<11.8, >44.7)
Cancer not smoking-related	99	1.19 (0.88, 1.60)	0.610	concave	32.7 (<11.8, >44.7)

Supplementary Table S10. Linear hazard ratios for all-cause and cause-specific mortality per 5 kg m^{-2} of BMI, with those missing smoking data excluded instead of being included as a separate class. BMI: body mass index, HR: hazard ratio, CI: confidence interval. BMI was measured at approximately age 20 and around 2001, when subjects were aged 49-78. Cox proportional hazards regression was used with age as the time axis. Models were adjusted for sex, date of birth (cubic splines) and smoking behaviour at the time of BMI reporting. Nonlinearity was assessed by adding BMI^2 to the model and assessing its coefficient's departure from the null ($P_{quadratic}$). BMI_{vertex} was estimated as the BMI at which the tangent to the quadratic model was horizontal. In a convex quadratic curve (coefficient for BMI^2>0), BMI_{vertex} estimates the BMI at which mortality is minimised. BMI_{vertex} values outside the observed range of BMI (11.8 to 44.7 kg m^{-2}) indicate a monotonically increasing or decreasing association among the observed data and were abbreviated for ease of presentation.

Exposure, cause of death	Deaths	HR (95% CI)	$P_{quadratic}$	Shape	BMI_{vertex} (95% CI)
BMI at age 20, follow-up from age 20 (N=10,768):					
All cause	2,347	1.04 (0.94, 1.14)	0.025	convex	21.6 (16.7, 24.1)
Cardiovascular disease	765	1.15 (0.98, 1.35)	0.376	convex	18.5 (<11.8, >44.7)
Respiratory disease	168	0.61 (0.42, 0.88)	0.976	convex	>44.7 (<11.8, >44.7)
Smoking-related cancer	289	0.90 (0.69, 1.17)	0.797	concave	16.5 (<11.8, >44.7)
Cancer not smoking-related	624	1.17 (0.98, 1.39)	0.490	convex	16.8 (<11.8, >44.7)
BMI in 2001, follow-up from 2001 (N=4,789):					
All cause	707	0.95 (0.85, 1.07)	0.001	convex	27.6 (25.4, 30.4)
Cardiovascular disease	229	1.10 (0.90, 1.34)	0.032	convex	25.6 (15.3, 29.6)
Respiratory disease	61	0.96 (0.64, 1.43)	0.547	convex	27.7 (<11.8, >44.7)
Smoking-related cancer	76	0.75 (0.52, 1.08)	0.936	concave	<11.8 (<11.8, >44.7)
Cancer not smoking-related	217	1.12 (0.92, 1.37)	0.760	concave	37.1 (<11.8, >44.7)
BMI at age 20, follow-up from 2001 (N=4,660):					
All cause	695	1.05 (0.88, 1.24)	0.455	convex	20.9 (<11.8, 44.3)
Cardiovascular disease	224	1.17 (0.87, 1.58)	0.506	convex	19.1 (<11.8, >44.7)
Respiratory disease	58	0.78 (0.41, 1.47)	0.586	concave	19.8 (<11.8, 41.2)
Smoking-related cancer	72	0.72 (0.41, 1.26)	0.756	concave	15.9 (<11.8, >44.7)
Cancer not smoking-related	214	1.04 (0.77, 1.41)	0.939	convex	16.0 (<11.8, >44.7)
BMI at age 20, follow-up from age 20, healthy non-smokers at age 20 (N=6,363):					
All cause	1,108	1.18 (1.04, 1.35)	0.051	convex	19.5 (<11.8, 26.1)
Cardiovascular disease	350	1.34 (1.06, 1.70)	0.315	convex	16.8 (<11.8, >44.7)
Respiratory disease	61	0.52 (0.28, 0.97)	0.906	concave	<11.8 (<11.8, >44.7)
Smoking-related cancer	104	1.64 (1.09, 2.46)	0.824	concave	>44.7 (<11.8, >44.7)
Cancer not smoking-related	354	1.21 (0.97, 1.52)	0.632	convex	14.8 (<11.8, >44.7)
BMI in 2001, follow-up from 2001, never-smokers in 2001 (N=2,226):					
All cause	256	1.02 (0.84, 1.25)	0.091	convex	26.1 (14.5, 35.6)
Cardiovascular disease	74	1.26 (0.88, 1.80)	0.419	convex	21.9 (<11.8, >44.7)
Respiratory disease	19	0.74 (0.34, 1.63)	0.976	concave	<11.8 (<11.8, >44.7)
Smoking-related cancer	17	1.00 (0.46, 2.14)	0.778	concave	26.0 (<11.8, >44.7)
Cancer not smoking-related	99	1.18 (0.87, 1.58)	0.648	concave	33.1 (<11.8, >44.7)

Supplementary Table S11. Linear hazard ratios for all-cause and cause-specific mortality per 5 kg m^{-2} of BMI at age 20, showing the effect of restriction to (i) healthy non-smokers, (ii) healthy subjects, and (iii) non-smokers, at age 20. BMI: body mass index, HR: hazard ratio, CI: confidence interval. Cox proportional hazards regression was used with age as the time axis. Models were adjusted for sex, date of birth (cubic splines) and smoking behaviour at age 20. Nonlinearity was assessed by adding BMI^2 to the model and assessing its coefficient's departure from the null ($P_{quadratic}$). BMI_{vertex} was estimated as the BMI at which the tangent to the quadratic model was horizontal. In a convex quadratic curve (coefficient for BMI^2>0), BMI_{vertex} estimates the BMI at which mortality is minimised. BMI_{vertex} values outside the observed range of BMI (11.8 to 44.7 kg m^{-2}) indicate a monotonically increasing or decreasing association among the observed data and were abbreviated for ease of presentation.

Exposure, cause of death	Deaths	HR (95% CI)	$P_{quadratic}$	Shape	BMI_{vertex} (95% CI)
BMI at age 20, follow-up from age 20 (N=11,233):					
All cause	2,438	1.04 (0.95, 1.14)	0.021	convex	21.5 (16.7, 23.7)
Cardiovascular disease	804	1.13 (0.96, 1.32)	0.416	convex	18.6 (<11.8, >44.7)
Respiratory disease	173	0.61 (0.42, 0.87)	0.859	concave	<11.8 (<11.8, >44.7)
Smoking-related cancer	298	0.92 (0.71, 1.19)	0.880	concave	14.3 (<11.8, >44.7)
Cancer not smoking-related	647	1.22 (1.03, 1.45)	0.377	convex	16.6 (<11.8, >44.7)
BMI at age 20, follow-up from age 20, healthy non-smokers at age 20 (N=6,363):					
All cause	1,108	1.18 (1.04, 1.35)	0.051	convex	19.5 (<11.8, 26.1)
Cardiovascular disease	350	1.34 (1.06, 1.70)	0.315	convex	16.8 (<11.8, >44.7)
Respiratory disease	61	0.52 (0.28, 0.97)	0.906	concave	<11.8 (<11.8, >44.7)
Smoking-related cancer	104	1.64 (1.09, 2.46)	0.824	concave	>44.7 (<11.8, >44.7)
Cancer not smoking-related	354	1.21 (0.97, 1.52)	0.632	convex	14.8 (<11.8, >44.7)
BMI at age 20, follow-up from age 20, healthy subjects only (N=9,206):					
All cause	1,876	1.07 (0.97, 1.19)	0.011	convex	21.2 (16.5, 23.0)
Cardiovascular disease	623	1.18 (0.99, 1.41)	0.307	convex	18.7 (<11.8, >44.7)
Respiratory disease	126	0.61 (0.40, 0.93)	0.404	concave	17.5 (<11.8, >44.7)
Smoking-related cancer	222	0.94 (0.69, 1.27)	0.502	convex	23.4 (<11.8, 44.5)
Cancer not smoking-related	504	1.21 (0.99, 1.46)	0.250	convex	18.8 (<11.8, >44.7)
BMI at age 20, follow-up from age 20, non-smokers only (N=7,663):					
All cause	1,421	1.09 (0.97, 1.23)	0.065	convex	20.6 (<11.8, 27.5)
Cardiovascular disease	447	1.23 (1.00, 1.52)	0.368	convex	17.6 (<11.8, >44.7)
Respiratory disease	81	0.45 (0.26, 0.78)	0.656	concave	<11.8 (<11.8, >44.7)
Smoking-related cancer	137	1.31 (0.90, 1.89)	0.604	concave	27.4 (<11.8, >44.7)
Cancer not smoking-related	444	1.20 (0.97, 1.47)	0.806	convex	<11.8 (<11.8, >44.7)

Supplementary Figure S1. Hazard ratios for all-cause and cause-specific mortality, relative to the first quartile, for sex-specific quartiles of body mass index (BMI) at age 20. Quartiles are plotted at their median values of BMI. Analyses were adjusted for sex, date of birth and smoking behaviour at age 20.

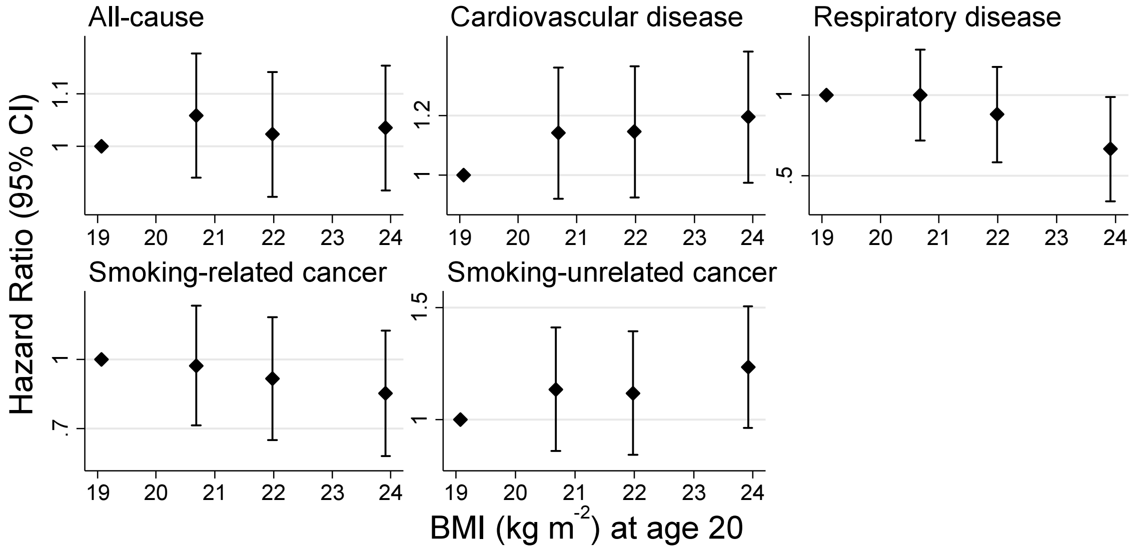

Supplementary Figure S2. Hazard ratios for all-cause and cause-specific mortality, relative to the first quartile, for sex-specific quartiles of body mass index (BMI) in 2001. Quartiles are plotted at their median values of BMI. Analyses were adjusted for sex, date of birth and smoking behaviour in 2001.

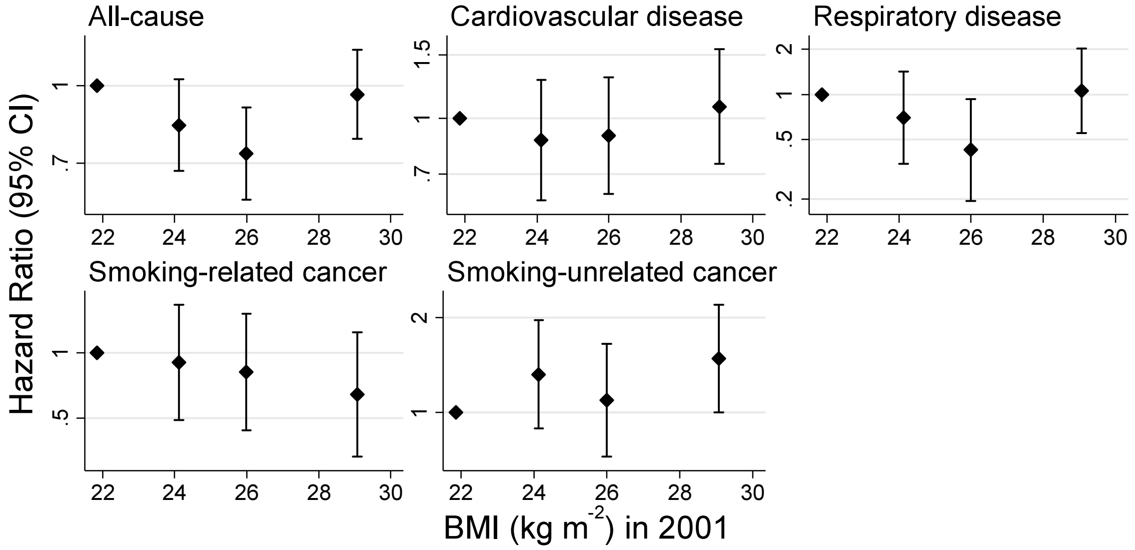

Supplementary Figure S3. Fitted hazard ratios for all-cause and cause-specific mortality from the quadratic model of BMI at age 20, including only those with BMI between the 5th and 95th percentiles. The midpoint of the recommended BMI range (18.5-25 kg m^{-2}) is used as the reference point. Analyses were adjusted for sex, date of birth and smoking behaviour at age 20.

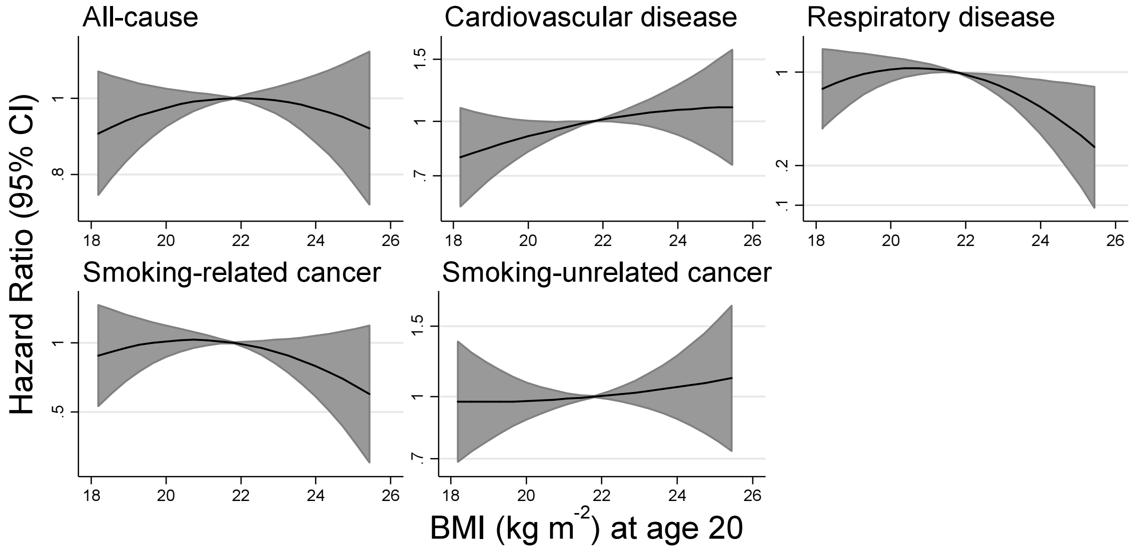

Supplementary Figure S4. Fitted hazard ratios for all-cause and cause-specific mortality from the quadratic model of BMI at age 20, with follow-up beginning after a 10-year lag. The midpoint of the recommended BMI range (18.5-25 kg m^{-2}) is used as the reference point. Analyses were adjusted for sex, date of birth and smoking behaviour at age 20.

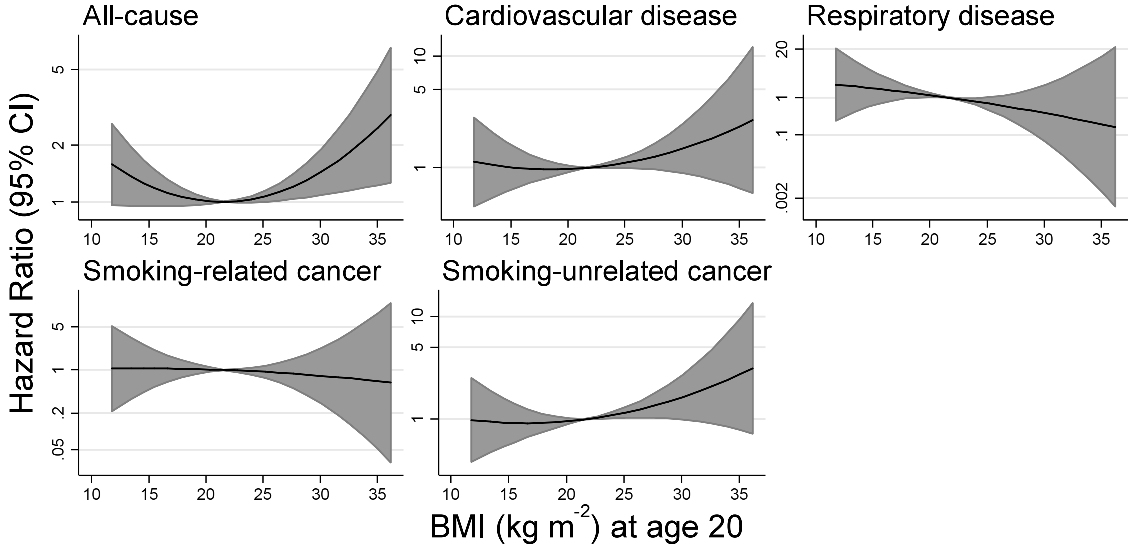

Supplementary Figure S5. Fitted hazard ratios for all-cause and cause-specific mortality from the quadratic model of BMI at age 20, with additional adjustment. The midpoint of the recommended BMI range (18.5-25 kg m^{-2}) is used as the reference point. Analyses were adjusted for sex, date of birth (cubic splines), smoking behaviour at age 20, father's social class, height (cubic spline), birth order, number of siblings, pulse rate and age at menarche.

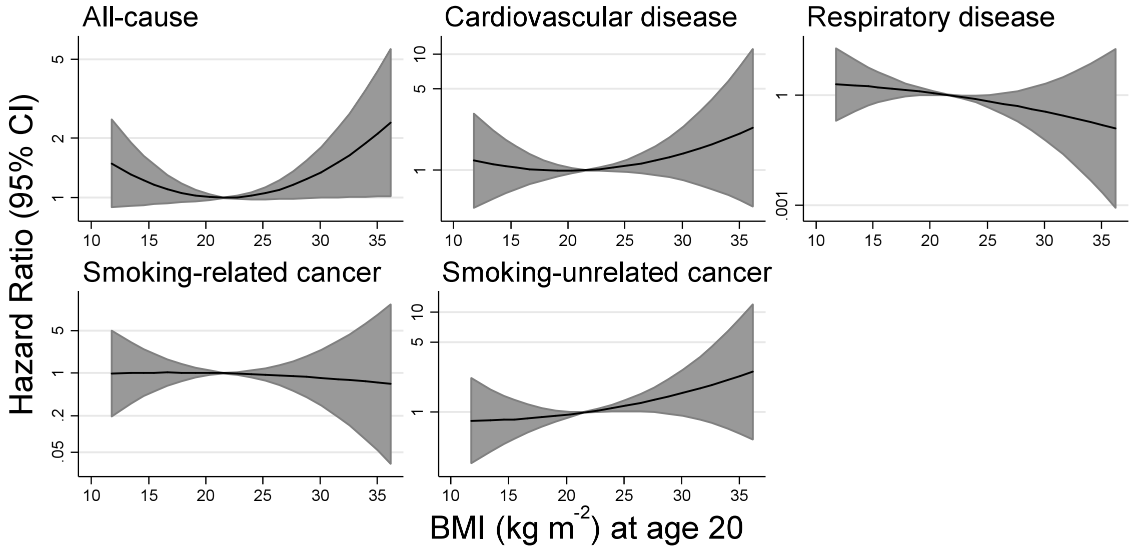

Supplementary Figure S6. Fitted hazard ratios for all-cause and cause-specific mortality from the quadratic model of BMI at age 20, with those missing smoking data excluded instead of being included as a separate class. The midpoint of the recommended BMI range (18.5-25 kg m^{-2}) is used as the reference point. Analyses were adjusted for date of birth and smoking behaviour at age 20.

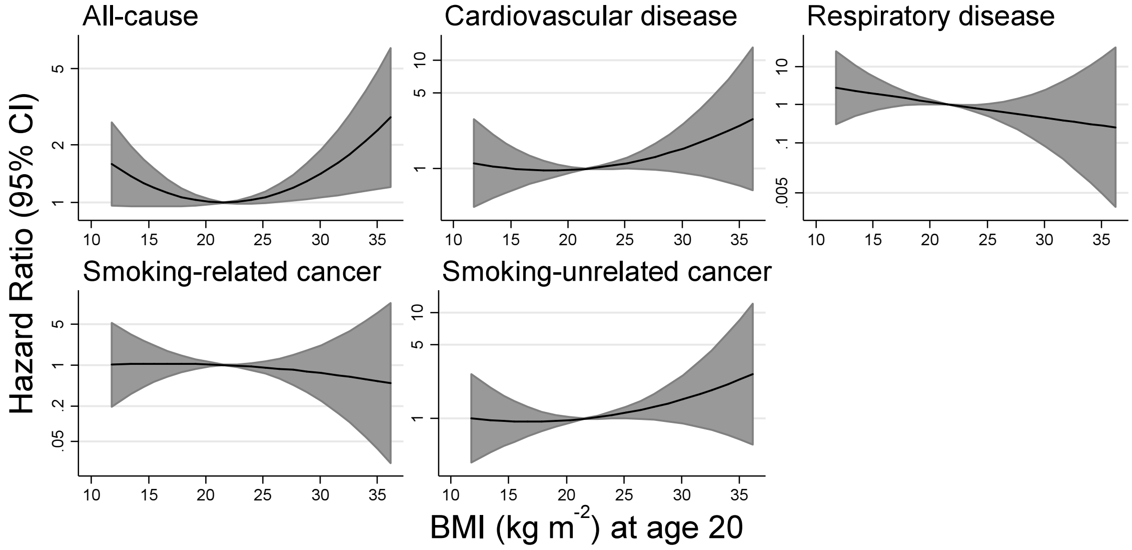

Supplement: Supplementary Information [file srep36046-s1.pdf]
